# Experiences from youth advisors in chronic disease prevention research

**DOI:** 10.1186/s40900-024-00585-z

**Published:** 2024-06-07

**Authors:** Dominik Mautner, Radhika Valanju, Imeelya Al Hadaya, Meera Barani, Alexi Cross, Emily McMahon, Bowen Ren, Dominique Rose, Aviral Sharda, Alexander Sinnett, Fulin Yan, Sara Wardak

**Affiliations:** 1 The Health Advisory Panel for Youth at The University of Sydney (HAPYUS), Faculty of Medicine and Health, The University of Sydney, Sydney, Australia; 2 Susan Wakil School of Nursing and Midwifery, Faculty of Medicine and Health, The University of Sydney, Level 6, Susan Wakil Building, Sydney, NSW 2006 Australia

**Keywords:** Youth, Adolescent, Participatory research, Advisory group, Consumer, Chronic disease prevention

## Abstract

Engaging young people in research is a promising approach to tackling issues like chronic disease prevention. Our involvement as youth advisors provided valuable experiences, including being at the forefront of change and learning to work within a research team. Furthermore, our experience provides greater insight and learnings for future youth engagement in research.

## Abstract (plain language summary)

We are a group of 16 diverse young people from New South Wales, Australia, who are passionate about youth health. In 2021 and 2022, we formed the Health Advisory Panel for Youth at the University of Sydney (HAPYUS, pronounced ‘Happy Us’) working with researchers on projects to prevent chronic diseases in young people. We brainstormed health issues from our own experiences and other research and summarised them into the top three youth health concerns. From these, we helped develop and test programs to support healthy behaviours in young people. We used scientific and public events to present our findings. Finally, we presented our results in a research paper and through traditional and social media. One of the most rewarding experiences was the opportunity to be part of all stages of the research process of improving youth health especially because COVID-19 and social media changed the way we need to think about youth mental and physical health. We also learned how to work together amongst ourselves as young people and within a research team. We hope that other young people can learn from our experiences and feel inspired to become active contributors in projects for meaningful change in the lives of young people.

## Comment

Chronic diseases in young people are rising at an alarming rate calling for research into possible preventative measures [1]. There is growing evidence that health outcomes in young people may be improved through interventions that are targeted and youth-specific, e.g., using youth-relevant language, technology, and motivational cues [2–5]. One promising approach is by including young people in appropriate research decision-making using a framework of collaboration informed by the Youth Participatory Action Research principles and processes and guidelines on adolescent participation and civic engagement [6–8]. As a result, the pilot Health Advisory Panel for Youth at the University of Sydney (HAPYUS, pronounced ‘Happy Us’) was established to enhance collaborations between young people and researchers as a vital step in improving current efforts in public health [9, 10]. We, the group of young people from HAPYUS, are co-authors on the paper “Striking the right balance: co-designing the Health4Me healthy lifestyle digital health intervention with adolescents,” [8] published in December 2023. We also participated in a formal evaluation of the youth advisory group, which has been published elsewhere and includes demographic details of our group and details regarding the process of establishing and facilitating the group [9]. We have written this comment as follow-up to the already published papers to provide a unique and different perspective as young people. As young people, we are often not meaningfully included in scientific discourse about research that affects us. Thus, we aim to provide additional learnings for future youth engagement in research.

Selected by researchers of the University of Sydney via an application process, HAPYUS started as a group of 16 young people aged between 13 and 18 years from across New South Wales Australia from diverse backgrounds and with different experiences, who had never met before but shared a passion for youth advocacy and youth health, and who were willing to commit time and effort into a shared endeavour despite the ongoing pandemic at the time. During 2021/22, we engaged in discussion through a hybrid model of virtual meetings, online chat discussions and in-person workshops to tackle the issues of improving youth’s physical and mental health. Our research and development process involved the following stages:

1. Conceptualising: brainstorming top health issues by drawing from young people’s lived experiences as well as relevant research literature.
2. Refining: distilling the broad range of issues into the top three health concerns and research questions [11].
3. Prototyping: shaping initiatives and digital programs to support healthy behaviours in young people such as the “Health4Me” program [8, 12] and “YES!” Project (unpublished).
4. Testing: scaling up of prototypes for use by adolescents; receiving feedback from participants as well as youth advisors.
5. Communicating: presenting our perspectives and recommendation strategies through a written report, media releases, and further distribution via social media platforms.

Working through a structured process enabled us as youth advisors to learn about various aspects of research and youth engagement. Guided by the three principles of Youth Participatory Action Research [13–15] we (a) worked inquiry-based, tackling questions that were grounded in our lived experiences, (b) participated in all steps of the research process and (c) aimed to change knowledge and practices to improve the lives of youth by active intervention. Furthermore, we gained valuable insights into collaboration and communication in different forms. While initial workshops and meetings were led by researchers, the project steps were subsequently divided into smaller tasks and worked on individually or in smaller groups, which self-selected based on interest or experience. Working remotely, we realised that to collaborate effectively we needed access to shared technology, efficient file-sharing, the possibility of co-authoring, video conferencing without time limits, and making use of our different strengths and experiences. As we mainly worked self-driven and asynchronous at different times of the day and at different days, we determined that feedback and planning is crucial as well as the ability to build on the work of others while also seamlessly moving in-between different tasks and different projects and research teams. Through continuous feedback amongst each other as well as regular update meetings with researchers we enhanced our efficiency as a team as well as our personal growth. We identified three main experiences as most valuable to us throughout the course of our roles at HAPYUS, (1) being at the forefront of change, (2) learning to work within a research team, and (3) communicating data and perspectives.

## Being at the forefront of change

As youth advisors, we share a sense of scientific curiosity and proactive engagement for health issues. Working with researchers from the Faculty of Medicine and Health at The University of Sydney enabled our group to be at the forefront of a rapidly evolving landscape of youth and adolescent preventive health research. We mainly focused on prevention efforts for chronic diseases such as cardiovascular diseases, type 2 diabetes and obesity. Risk factors for these chronic diseases are often established in younger years, but prevention programs are mostly geared towards adults [16, 17]. In past generations, this approach might to some extent have been effective for younger people as well, as most age groups were exposed to similar communication channels, such as print, TV, billboards, or radio. However, with the rise of digital technology, social media, and personalised algorithms, many young people are now using different media than adults and ‘traditional’ prevention campaigns often do no longer reach younger age groups [18]. Youth engagement provides an avenue for researchers to collaborate directly with young people as members of the research team to conduct prevention research that is relevant to young people, using their preferred platforms. Those efforts seem especially relevant for preventable chronic diseases, as they pay a triple dividend by improving the lives of young people in the present, but also by providing them with a chance to lead a full adult life and subsequently giving rise to future generations.

Having identified three top concerns regarding chronic disease prevention in young people (i.e., social media’s inimical impact on young people, unbalanced nutritional intake, and rise of physical inactivity) [11], we realised that these issues were dynamically changing based on the multidimensional context of COVID-19. This also included growing concerns for youth mental health with research suggesting a worsening of social isolation and psychological distress [19, 20], particularly associated with depression and anxiety [21, 22]. We understood that existing concepts about youth mental and physical health needed modification due to restrictions posed by the pandemic with not only disruptions to daily life, but also loss of support and services outside the family home leading to an increase in demand of services but simultaneously to constraints on the supply of those services [21–24]. On the other hand, new opportunities in service delivery emerged with the rising prevalence of social media that could be employed as alternative avenue of information and service delivery. The challenge was to create content that is relevant for the target audience. Being part of the same age bracket and with diverse and lived experiences our group was uniquely positioned to reach that target group through content creation of topics on chronic disease prevention. Discussing these concerns with researchers gave us the opportunity to represent the perspective of young people whilst also collaborating on new ways to overcome adolescent health issues, such as with the following projects and our reflections presented in Table 1.

**Table 1:** Reflections on our involvement in adolescent health research projects

Project	Our reflections
Health4Me: digital health program to support young people’s nutrition and physical well-being	By considering the ever-growing presence of technology in the daily lives of youth, we recognised text messaging as a high yield avenue for reaching out to younger audiences. Using the identified top health issues, we collectively brainstormed text messages that would remind and encourage young people to complete short, health-related activities, to help improve their physical and mental health. By providing our input, we were able to construct text messages that we as young people felt would resonate with other teenagers and at the same time achieve potentially health-relevant modifications in behaviour. This represents a significant shift in conventional adolescent health research approaches by increasing youth involvement in the creation of meaningful health tools (“by youth, for youth”). The study protocol and co-design process for this study have been published and our contribution is acknowledged [8, 12].
YES! Project Youth Engagement Study	Another oftentimes limiting aspect of current research regarding youth health is lack of feedback from young people on data collected about them. Thus, our collaboration on this project emphasised the analysis of youth responses, such as real-time viewpoints from youth on questions about civic engagement at all levels. Additional opportunities for us were also provided in shadowing focus groups by scribing key issues explored by participants. Thus, this project emphasised youth involvement at every stage through a multitude of roles for young people, from collaborators to participants, to effectively improve the feedback loop that shapes our understanding of perceptions of youth engagement. This project is ongoing, and we look forward to collaborating on the scientific manuscript.
Youth Advisory Group (YAG) Evaluation Study	Thirteen members of HAPYUS participated in an evaluation study that consisted of completing a written and verbal questionnaire about our experiences as part of HAPYUS in intervals of 6 months [9]. This is an innovative approach to embed youth feedback into research studying the impact on youth collaborators, who are designing health tools, rather than studying the target group for the health tools (which is young people using the health applications). Additionally, reflecting on our communication, teamwork skills and confidence, most of us agreed that our research engagement helped us learn more about our holistic development as individuals.

## Working within a research team

Despite geographic distance and COVID-19 restrictions, we were able to collaborate within our advisory team as well as with the team of researchers via online platforms such as Slack and Mural. The online environment allowed us to contribute equally independent of location and scheduling constraints. Shared access and building on each other’s work meant being open and tolerant to constantly changing and evolving files and accept some loss of ‘ownership’ of individual contributions as projects evolved, but this evolved into shared ownership which was empowering. Working online also meant missing out on some shared experiences beyond the immediate tasks at hand. On the other hand, our mutual learning and increased efficiency meant we improved our work on many levels. The diversity of our group, both culturally and geographically, proved valuable in identifying the complex interactions leading to chronic disease in young people including barriers to healthy living. We were able to draw on our lived experience and share these experiences with the group, if we felt comfortable. For example, group members from urban areas reported in general better access to health services, whereas some group members from rural communities had the advantage of a broader network of family and neighbours mitigating the effects of sudden loss of social contacts during the pandemic. Also, we discussed how ethnicity and gender stereotypes played a role in reduced sport participation and how health information was communicated on social media. To effectively delegate responsibilities based on strengths and interests, we divided ourselves into working teams and subsequently updated other working teams through a regular feedback system.

Within the HAPYUS advisory group, the team of co-chairs worked together on collating the experiences of all members culminating in a report and media releases, a process that involved regular regrouping. We also formed teams for consulting with the various state and federal parliamentarians, speaking at the Australian Medical Association conference, and for drafting policy briefings. As a team with sixteen members, it was crucial that we distributed opportunities equally amongst ourselves to allow everyone to be involved in collaborations on both a small and large scale.

The most valuable aspect of working within the Youth Participatory Action Research framework was the guidance of researchers which motivated us for constant improvement using feedback in areas of delegation and time management to ensure we worked together effectively. Learning those skills will also be important for the future, as in a rapidly changing workforce, online collaboration is expected to be an essential skill.

## Communicating data and perspectives

Traditionally, young people have been distanced from both readership of academic literature (due to jargon that may be difficult for readers from non-research backgrounds and with limited awareness of relevant materials available regarding youth health issues) as well as co-authorship of academic literature (as research was considered strictly a domain of adult experts). HAPYUS represented to us as youth advisors an innovative path of youth involvement allowing us to use our own voice. To avoid tokenistic youth participation, we felt the need to create an authentic account that would be accessible to younger audiences throughout the world. Our group co-authored a perspective essay about the evolution of youth health contextualised by the pandemic, which was published by The Lancet Child and Adolescent Health as “Youth perspective on chronic disease prevention” [11]. As some of the youngest co-authors to be published in The Lancet, we achieved two important goals:

- Bridging the gap between youth and academia.
- Presenting our concerns through synthesising research evidence and perspectives, leading to increased awareness of chronic disease in young people and potentially novel approaches for solutions.

Through the ensuing interest by national level media, we had an opportunity for further public discussions, such as through The Sydney Morning Herald (national Australian newspaper) and Sunrise (national Australian breakfast television program), raising awareness amongst the wider scientific community but also the public including younger audiences. Other opportunities included presenting at the Australian Medical Association Conference (the peak professional body for medical doctors in Australia), and discussions with policy advisors of the state and federal Government.

Data communication is becoming an increasingly sought-after skill and we as youth advisors greatly benefited from working through various modes of communication, from article writing to interviews and conference presentations. Through these opportunities, we gained invaluable experiences, but most importantly, we hopefully contributed in a meaningful way to youth health research and better health outcomes for young people.

## Conclusions

In conclusion, our involvement in the HAPYUS has been influential in advancing youth engagement in chronic disease prevention research. Based on the principles of Youth Participatory Action Research we navigated dynamic health challenges, improved our collaboration skills, and bridged the gap between youth and researchers through effective communication. Our experiences offer valuable insights for future youth involvement in addressing chronic disease prevention research.

## Data Availability

No datasets were generated or analysed during the current study.

## Abbreviations

HAPYUS: Health Advisory Panel for Youth at the University of Sydney
